# Diagnosis of Chronic Infection at Total Hip Arthroplasty Revision Is a Question of Definition

**DOI:** 10.1155/2021/8442435

**Published:** 2021-11-19

**Authors:** Sebastian Philipp Boelch, Kilian Rüeckl, Laura Elisa Streck, Viktoria Szewczykowski, Manuel Weißenberger, Axel Jakuscheit, Maximilian Rudert

**Affiliations:** Department of Orthopaedic Surgery, Julius-Maximilians University, Koenig-Ludwig-Haus, Brettreichstrasse 11, 97074 Wurzburg, Germany

## Abstract

**Purpose:**

Contradicting definitions of periprosthetic joint infection (PJI) are in use. Joint aspiration is performed before total hip arthroplasty (THA) revision. This study investigated the influence of PJI definition on PJI prevalence at THA revision. Test quality of prerevision aspiration was evaluated for the different PJI definitions.

**Methods:**

256 THA revisions were retrospectively classified to be infected or not infected. Classification was performed according to the 4 different definitions proposed by the Musculoskeletal Infection Society (MSIS), the Infectious Diseases Society of America (IDSA), the International Consensus Meeting (ICM), and the European Bone and Joint Infection Society (EBJIS). Only chronic PJIs were included.

**Results:**

PJI prevalence at revision significantly correlated with the applied PJI definition (*p* = 0.01, Cramer's *V* = 0.093). PJI prevalence was 20.7% for the MSIS, 25.4% for the ICM, 28.1% for the IDSA, and 32.0% for the EBJIS definition. For synovial fluid white blood cell count, the best ROC-AUC for predicting PJI was 0.953 in combination with the MSIS definition.

**Conclusion:**

PJI definition significantly influences the rate of diagnosed PJIs at THA revision. Synovial fluid white blood cell count is a reliable means to rule out PJI. In cases with a borderline high synovial white blood cell count before THA revision as the only sign of chronic PJI, an extended diagnostic work-up should be considered.

## 1. Introduction

Periprosthetic joint infection (PJI) has become a rare complication in primary total hip arthroplasty (THA), occurring in 0.2% to 2.0% [1, 2]. However, because the absolute number of THA revision is increasing, the number of PJIs will increase, too [3].

Since the eminent review on PJIs by Zimmerli et al. in 2004 [4], a number of PJI definitions have been proposed. The Musculoskeletal Infection Society (MSIS) was the first to publish a standard for the definition of PJI in 2011. This definition inaugurated the major criteria of PJI, which were the presence of a sinus tract or repeated pathogen isolation from the periprosthetic surrounding [5]. This definition was followed by a guideline published by the Infectious Diseases Society of America (IDSA) in 2012. Herein, pathologic periprosthetic tissue results and purulence were added to the pathognomonic major criteria of PJI [6]. The International Consensus Meeting (ICM), which was held in 2013 and in 2018, introduced a likelihood scale calculated from the presence of major and also of minor criteria [7]. The European Bone and Joint Infection Society (EBJIS) lately added the synovial white blood cell count (sWBC) as a major PJI criterion in their proposal for a PJI definition [8]. But still, the consensus on what defines PJI is weak [7]. Particularly, the biofilm-associated subtypes, i.e., chronic PJIs, are causing trouble being defined. Additionally, the clinical presentation of chronic infection typically is subliminal [7, 8]. Prerevision recognition of chronic PJI is of high medical, legal, and economical significance, because the treatment concepts of septic and aseptic THA failures are dissimilar. Thus, diagnostic algorithms advocate prerevision aspiration to exclude chronic PJI as an unrecognized cause of THA failure before revision [9–11].

Therefore, this study aimes to invistigate whether the choice of PJI definition influences the prevalence of PJIs at THA revision. Additionally, the test quality of prerevision aspiration was evaluated for the different PJI definitions.

## 2. Materials and Methods

### 2.1. Patients

Approval for this retrospective study was given by the institution's ethics committee (Reference number 2016072801). 349 cases that underwent THA revision with collection of at least 2 tissue samples for culturing and with anteceding joint aspiration between January 2012 and December 2019 were retrospectively identified. 22 cases were excluded due to a punctio sicca and 18 because the indwelling prosthesis was an interim spacer. Further, 47 patients with symptoms lasting less than 3 weeks and 6 patients that received antibiotics within 14 days before aspiration were excluded. Thus, 256 THA revision cases were included in this study.

### 2.2. Evaluation of Periprosthetic Joint Infection

At the study institution, PJI is evaluated on the basis of patients' history, clinical findings as well as blood infection parameters, imaging studies, and polymerase chain reaction-based means in particular situations. THAs with a chronic history of complaints are aspirated by default to rule out PJI before proceeding to revision surgery. Synovial fluid is cultivated for at least 14 days. With the remaining aspirate, synovial fluid white blood cell count (sWBC) was performed. During revision surgery, 2 to 5 tissue specimens are collected and cultured for 14 days. Tissue samples are taken and evaluated for failure reason by a pathology specialist.

### 2.3. Definitions of Periprosthetic Joint Infection

All 256 revised THAs were retrospectively classified as PJI or no PJI. Classification was performed according to the definitions issued by the MSIS in 2011, by the IDSA in 2013, and by the EBJIS in 2021. Since the ICM discriminates an acute from a chronic situation, only the criteria for the chronic PJI were taken into account. For the MSIS criteria, PJI can be either confirmed, if at least one of the major criteria or if at least 4 of the 6 minor criteria are present. In contrast, the ICM definition comprises a scoring system based on major and minor criteria. PJI is defined by either at least one major criterion or if the scoring of the minor criteria sums up to at least 6 points. With the IDSA and the EBJIS criteria, PJI is confirmed by the presence of at least one major criterion.  Table 1 summarizes the applied definitions.

### 2.4. Diagnostic Value of Prerevision Joint Aspiration

Synovial fluid culture results and sWBC results from prerevision aspiration were compared with the 4 different PJI definitions. Sensitivity, specificity, and positive (PPV) and negative predictive values (NPV) were calculated in comparison to the applied definition. For sWBC, the test quality was additionally evaluated.

### 2.5. Statistics

Data is displayed as mean and range. Correlation between PJI definitions and PJI rate was evaluated with the chi^2^ test. Significance was set at *p* < 0.05. The effect power was calculated with Cramer's *V*. Receiver operating characteristic (ROC) curves were plotted for sWBC depending on the applied definition. The area under the curve (ROC-AUC) analysis was performed to quantify the quality of the test. Statistics were performed with SPSS 28 (SPSS Inc., USA).

## 3. Results

### 3.1. Patients

52.0% (*N* = 133) of the 256 revisions were performed in female patients. Mean age was 69 years (20-90); the mean duration from aspiration to revision was 51 days (0-289). 63.6% (*N* = 163) had no previous revision, 30.9% (*N* = 79) had 1 to 3 previous revisions, and 5.5% (*N* = 14) had more than 3 previous revisions. Mean duration from previous surgery to revision was 95.6 months (2.2-406.6). 73.8% (*N* = 189) were cementless implants. At least one of the implant components was loose in 75.4% (*N* = 193). Mean C-reactive protein level at revision was 1.5 mg/dl (0.0-27.2).

### 3.2. Rate of PJI according to the Applied Definitions

Selection of PJI definition significantly correlated with the PJI rate (*p* = 0.03). However, the correlation was low (Cramer's *V* = 0.093). The distribution of PJI rates according to the applied definitions is shown in Table 2.

### 3.3. Diagnostic Value of Joint Aspiration before Revision

Table 3 shows sensitivity, specificity, PPV, and NPV for synovia fluid cultures from prerevision aspiration according to the applied PJI definition.

SWBC from prerevision aspiration was performed in 46.5% of the included revision cases (119/256).  Table 4 shows sensitivity, specificity, PPV, and NPV for sWBC according to the applied PJI definition.

**Figure 1:**
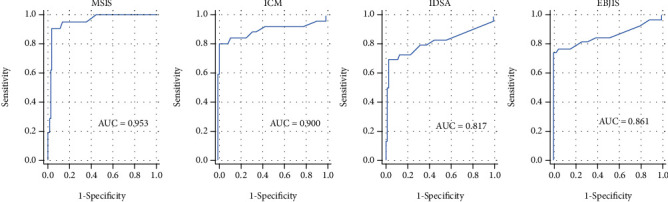
ROC-AUC analysis for the sWBC in comparison to the applied definitions. MSIS: Musculoskeletal Infection Society; ICM: International Consensus Meeting; IDSA: Infectious Diseases Society of America; EBJIS: European Bone and Joint Infection Society.

ROC-AUC analysis showed the best quality for sWBC as a predictor for PJI in combination with the MSIS definition (AUC = 0.953) followed by the combination with the ICM definition (AUC = 0.900) as depicted in Figure 1.

**Figure 2:**
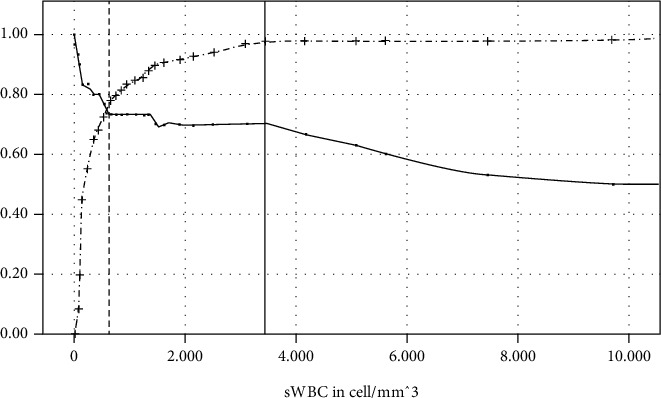
Sensitivity (continuous spline) and specificity (dotted spline) depicted as decimal (*y*-axis) in dependence of sWBC (*x*-axis). The vertical dotted line marks a sWBC of 650 cells/mm^3^ and the continuous line a sWBC of 3450 cells/mm^3^.

Because the IDSA definition is the only one that does not include the sWBC, the sensitivity and specificity calculated by ROC are exemplified.  Figure 2 shows that the sensitivity looses 3 percentage points from a sWBC of 650 cells/mm^3^ to 3450 cells/mm^3^ but specificity increases by 19.8 percentage points.

## 4. Discussion

The consensus on the definition of PJI is weak [7]. Several different PJI definitions have been established. Under this aspect, the current study investigated whether the selection of PJI definition influences the prevalence of chronic PJIs at THA revision. We found that PJI prevalence significantly correlated with the applied definition. Thus, chronic infection at THA revision is a question of definition. This conclusion needs to be considered whenever treatment decisions are made.

Although the correlation between PJI definition and PJI prevalence was significant, the association was low. All 53 revision cases classified as PJI by the MSIS definition were consistently classified a PJI by all of the 3 other definition systems. In contrast, no case was classified PJI exclusively by the ICM criteria. Further, 2 cases were classified PJI exclusively by the IDSA because purulence was the only indicator of PJI. Finally, 2 cases were classified PJI exclusively by the EBJIS because a sWBC > 3000 cells/mm^3^ was the only indicator of PJI. In these doubtful cases, treatment decision should include balancing probabilities carefully. This applies particularly for chronic PJIs, where the gold standard treatment is the two-stage exchange [12]. The 90-day mortality of the two-stage exchange has been reported to be 4%, and up to 10.5% of the patients are unfit to proceed to the second stage [13, 14]. Thus, the invasiveness necessary for infection eradication should be weighed against the risk of overtreatment.

Currently, joint aspiration with sWBC is considered the most important pillar to exclude PJI before revision [15, 16]. Irrespectively of the applied definition, sWBC proved to be of higher diagnostic value than synovial fluid culture. Comparable results are described in the review by Ahmad et al. In their study, sWBC had a sensitivity of 88% while synovial fluid culture had a sensitivity of only 72% [17]. However, the authors also stated a 95% confidence interval ranging from 0.81 to 0.90 for sWBC sensitivity and highlighted the issue of a lacking gold standard [17]. The current study compared the sWBC sensitivities when the 4 most important PJI definitions are used. The ROC-AUC analysis showed that the sWBC provides the best test quality in combination with the MSIS definition. At a cut-off of 3000 cells/mm^3^, the sensitivity was 90.5%. A trend towards lower cut-off values, for instance 1500 cells/mm^3^, can currently be observed in the literature [18–20]. However, Figure 2 shows that improving the sensitivity by lowering cut-off values goes along with enlarging the risk of overtreatment due to inadequate specificity. The herein presented data underline that even for chronic PJI, the sWBC with a cut-off of 3000 cells/mm^3^ should be appreciated a reliable threshold to rule out infection before THA revision. Nevertheless, sWBC may rather be considered a “guide value” rather than a “cut-off value.” If borderline high sWBC values are the only sign of infection, additional work-up to confirm PJI should be considered before proceeding to a two-stage exchange.

Due to its retrospective design, there are limitations to this study. Intraoperative tissue samples for histologic work-up were inconsistently evaluated. Rather than describing the sole number of neutrophilic granulocytes per high-power field (HPF), histologic findings were documented as to be confirmative for PJI or were classified according to Krenn et al. [21]. However, just as the definition of PJI shows a dynamic evolution so does histologic evaluation: The MSIS highlighted the fact that “histologic examination (...) may be operator dependent” but recommended a cut-off of 5 neutrophils per HPF [5]. The IDSA leaves the histologic definition up to the pathologist [6]. Last, while the ICM 2018 summary discusses the thresholds of at least 5 neutrophils in contrast to 10 neutrophils in each of 5 HPFs, the EBJIS criteria demand at least 23 neutrophils per 10 HPFs [16].

A further limitation is that sWBC was available in only 46.5% of the 256 included revision cases. However, within these selected cases, PJI prevalence was 17.7% for the MSIS, 21.8% for the ICM, 24.4% for the IDSA, and 31.1% for the EBJIS definition, respectively. Thus, prevalence of the cases with available sWBC was similar to the overall PJI prevalence. While at the study institution, synovia fluid samples were preferably used for pathogen identification before revision in the past, joint aspiration with sWBC now is performed to exclude PJI.

## 5. Conclusion

Orthopedic surgeons need to be aware that their choice of PJI definition significantly influences the rate of diagnosed PJIs at THA revision. Synovial white blood cell count is a reliable means to rule out PJI. In cases with a borderline high synovial white blood cell count before THA revision as the only sign of chronic PJI, an extended diagnostic work-up should be considered.

## Figures and Tables

**Table 1:** PJI definitions sorted by publishing organization.

MSIS [5]	IDSA [6]	ICM [7] (chronic)	EBJIS [8]
*Major criteria*	*Major criteria*	*Major criteria*	*Major criteria*
Sinus tract	Sinus tract	Sinus tract	Sinus tract
Pathogen isolated from at least two samples from synovia or periprosthetic tissue	Same pathogen isolated from at least two samples from synovia or periprosthetic tissue	Same pathogen isolated from at least two samples from the prosthesis' surrounding	Same pathogen isolated from at least two samples from the prosthesis' surrounding
*Minor criteria* (i) ESR > 30 mm/h and CRP >1 mg/dl (ii) Elevated sWBC (>3000 cells/mm^3^) (iii) Elevated PMN percentage in sWBC (iv) Presence of purulence (v) One pathogen isolated from prosthesis surrounding (vi) 5 neutrophils/HPF in histology^∗^	Growth of a virulent pathogen in a single specimen	*Minor criteria* (i) CRP > 1 mg/dl or D − dimer > 860 *μ*g/l. (ii) ESR > 30 mm/h (iii) sWBC > 3000/*μ*l or LE strip (++) or positive alpha defensin test (iv) >70% PMN in sWBC (v) Single positive culture (vi) Positive histology^∗^ (vii) Presence of purulence	Positive immunoassay or lateral flow assay
	Presence of purulence		sWBC > 3000/*μ*l
			>80% PMN
	Acute inflammation at histologic examination of periprosthetic tissue^∗^		Presence of ≥5 neutrophils in ≥5 HPFs or presence of visible microorganisms


^∗^For retrospective classification, infection was defined as judged by the pathologist. CRP: C-reactive protein; sWBC: synovial white blood cell count; PMN: polymorphonuclear neutrophils in synovia; HPF: high-power filed; LE: leucocyte esterase.

**Table 2:** Distribution of PJI according to the applied definition (*N* = 256). The rate for repeated pathogen detection, single pathogen detection, no pathogen detection based on prerevision aspiration, and intraoperative tissue samples is shown.

	MSIS % (*N*)	ICM % (*N*)	IDSA % (*N*)	EBJIS % (*N*)
PJI rate	20.7 (53)	25.4 (65)	28.1 (72)	32.0 (82)
Rate of repeated pathogen detection	98.1 (52)	75.4 (49)	68.1 (49)	57.0 (49)
Rate of single pathogen detection	1.9 (1)	13.9 (9)	13.9 (10)	19.8 (17)
Rate of PJI without pathogen detection	0.0 (0)	10.8 (7)	18.1 (13)	19.5 (16)

MSIS: Musculoskeletal Infection Society; ICM: International Consensus Meeting; IDSA: Infectious Diseases Society of America; EBJIS: European Bone and Joint Infection Society.

**Table 3:** Sensitivity, specificity, and positive and negative predictive values for synovia fluid cultures from prerevision joint aspiration according to the applied PJI definition in %.

	MSIS	ICM	IDSA	EBJIS
Sensitivity	67.9	58.5	51.4	52.4
Specificity	96.6	97.4	96.7	100.0
PPV	83.7	88.4	86.1	100.0
NPV	92.0	87.3	83.6	81.2

MSIS: Musculoskeletal Infection Society; ICM: International Consensus Meeting; IDSA: Infectious Diseases Society of America; EBJIS: European Bone and Joint Infection Society; PPV: positive predictive value; NPV: negative predictive value.

**Table 4:** Sensitivity, specificity, and positive and negative predictive values for sWBC from preoperative joint aspiration according to the applied PJI definition in %.

	MSIS	ICM	IDSA	EBJIS
Sensitivity	90.5	81.5	70.0	61.5
Specificity	95.2	98.0	96.7	100.0
PPV	82.6	95.7	91.3	100.0
NPV	97.9	95.8	91.7	85.4

MSIS: Musculoskeletal Infection Society; ICM: International Consensus Meeting; IDSA: Infectious Diseases Society of America; EBJIS: European Bone and Joint Infection Society; PPV: positive predictive value; NPV: negative predictive value.

## Data Availability

The datasets used and analyzed during the current study are available from the corresponding author on reasonable request.
